# Monitoring pilot trainees’ cognitive control under a simulator-based training process with EEG microstate analysis

**DOI:** 10.1038/s41598-024-76046-0

**Published:** 2024-10-20

**Authors:** Mengting Zhao, Wenjun Jia, Sion Jennings, Andrew Law, Alain Bourgon, Chang Su, Marie-Hélène Larose, Hugh Grenier, David Bowness, Yong Zeng

**Affiliations:** 1 Concordia Institute for Information Systems, Gina Cody School of Engineering and Computer Science, Concordia University, Montreal, Canada; 2 National Research Council of Canada, Aerospace Research Centre, Ottawa, Canada; 3 CAE Inc., St-Laurent, Canada; 4 Marinvent Corporation, St-Bruno, Canada

**Keywords:** Cognitive control, Pilot training, EEG analysis, EEG microstates, Neurophysiological measures, Decision, Navigation, Data processing

## Abstract

The objective of pilot training is to equip trainees with the knowledge, judgment, and skills to maintain control of an aircraft and respond to critical flight tasks. The present research aims to investigate changes in trainees’ cognitive control levels during a pilot training process while they underwent basic flight maneuvers. EEG microstate analysis was applied together with spectral power features to quantitatively monitor trainees’ cognitive control under varied flight tasks during different training sessions on a flight simulator. Not only could EEG data provide an objective measure of cognitive control to complement the current subjective assessments, but the application of EEG microstate analysis is particularly well-suited for capturing rapid dynamic changes in cognitive states that may happen under complex human activities in conducting flight maneuvers. Comparisons were conducted between two types of tasks and across different training stages to monitor how pilot trainees’ cognitive control responds to varied flight task types and training stages. The present research provides insights into the changes in trainees’ cognitive control during a pilot training process and highlights the potential of EEG microstate analysis for monitoring cognitive control.

## Introduction

Training and simulation technologies in aviation are moving towards more human-centered solutions, where pilots’ cognitive states can be inferred from physiological measures such as EEG, fNIRS, and ECG. There are successful applications where the physiological signals from pilots or drivers of vehicles were collected and assessed as indicators of cognitive changes. Among those physiological signals, EEG has attracted the most research interest^1–5^, as an appropriate tool for investigating the temporal changes in trainees’ brains without additional workload or interference. For example, research findings have linked alpha desynchronization to demands on attentional resources^6,7^. Decreases in alpha power were observed with increasing cognitive workload^8–10^, with significant alpha decreases found in the fronto-central and parietal regions^11,12^. Meanwhile, theta oscillations may support working memory^13–16^, cognitive control^17^, and rhythmic shifts of spatial attention^18^ as reviewed by (Herweg et al., 2020)^19^. Increases in EEG theta power were reported to be positively related to successful information encoding and memory retrieval during memory tasks^20–22^. Research findings also associated increases in theta activity with increased top-down control for attention allocation and information processing^23,24^, identifying certain brain areas involved during tasks requiring cognitive control, with the active involvement of the medial prefrontal cortex (mPFC) highlighted^25^. In particular, increases in frontal midline theta were observed under tasks with higher demands on cognitive control^26–28^.

Cognitive control refers to the ability to coordinate mental resources towards goal-directed performance while suppressing irrelevant distractions^29^. This function is critical for supporting the high cognitive demands of flying tasks, but it has not been as thoroughly investigated in the literature as other cognitive functions, such as cognitive workload, particularly in the context of pilot training^3,30–32^. The study of Roberts and colleagues^33^ reported the associated differences in frontal and parietal white matter under different cognitive control levels that were measured by two behavioral paradigms, namely the Eriksen Flanker^34^ and change of plan tasks^35^. In the study (Borghini et al., 2017)^3^, EEG spectral features were analyzed in responding to different cognitive control levels that were represented by the difficulty levels of three kinds of events, namely Skill, Rule, and Knowledge-based the SRK model proposed by Rasmussen^36^. However, these studies either indirectly measured cognitive control using behavioral performance^33,37^ or treated cognitive control as an independent variable^3,38^, which could reduce reliability or interfere with the original tasks. In contrast, the present research aimed to monitor trainees’ cognitive control changes via EEG features under different types of flight tasks during various training stages, without interfering with the training process by inserting additional events or interruptions.

EEG microstate analysis is an attractive method for simultaneously investigating the spatial properties and temporal dynamics of the human brain, but it has not been well used in aviation research. Unlike traditional EEG features, microstates capture scalp potential topography patterns and their changes over time^39,40^. Each microstate reflects an activation pattern in large-scale brain networks and represents a quasi-stable state of the brain^39^. As reviewed by (Michel and Koenig, 2018)^40^, the four microstates (A, B, C, D) identified in most previous studies exhibited high similarity, and further research has focused on the associated functional role. For instance, microstate D has been associated with the dorsal attention network^41,42^, and microstate C has been found to reflect activities in the default mode network (DMN) supported by EEG and fMRI evidence in the literature^42–44^. In this study, we investigated pilot trainees’ cognitive control using EEG microstate analysis under the neurophysiological context, which considers the brain’s intrinsic spatial and temporal dynamics. To this end, we applied EEG microstate analysis as the main method together with spectral power features to explore cognitive control variations in trainees under different training task types and stages within a pilot training program.

We hypothesized that trainees’ cognitive control would vary during a pilot training process in responding to differences in flight task types and training stages. Enhanced cognitive control has been associated with improvements in information processing, reasoning, planning, and decision-making^45,46^, all of which can occur during a learning process like pilot training^47,48^. Therefore, we conducted paired comparisons between two types of tasks, namely Baseline tasks and Trial tasks, within the same stage to investigate the variations of cognitive control under those two task types, which could also align our results with the effect of varied task demands and difficulties on cognitive control reported in the literature. Moreover, we analyzed pilot trainees’ EEG signals collected only under the baseline task type to assess and compare their cognitive control levels when they were requested to repeat the same maneuvers as the training continued. Finally, we compared pilot trainees’ cognitive control variations as indicated by EEG features with the subjective evaluations provided by experienced training instructors.

## Materials and methods

### Participants and experiment procedure

**Fig. 1:**
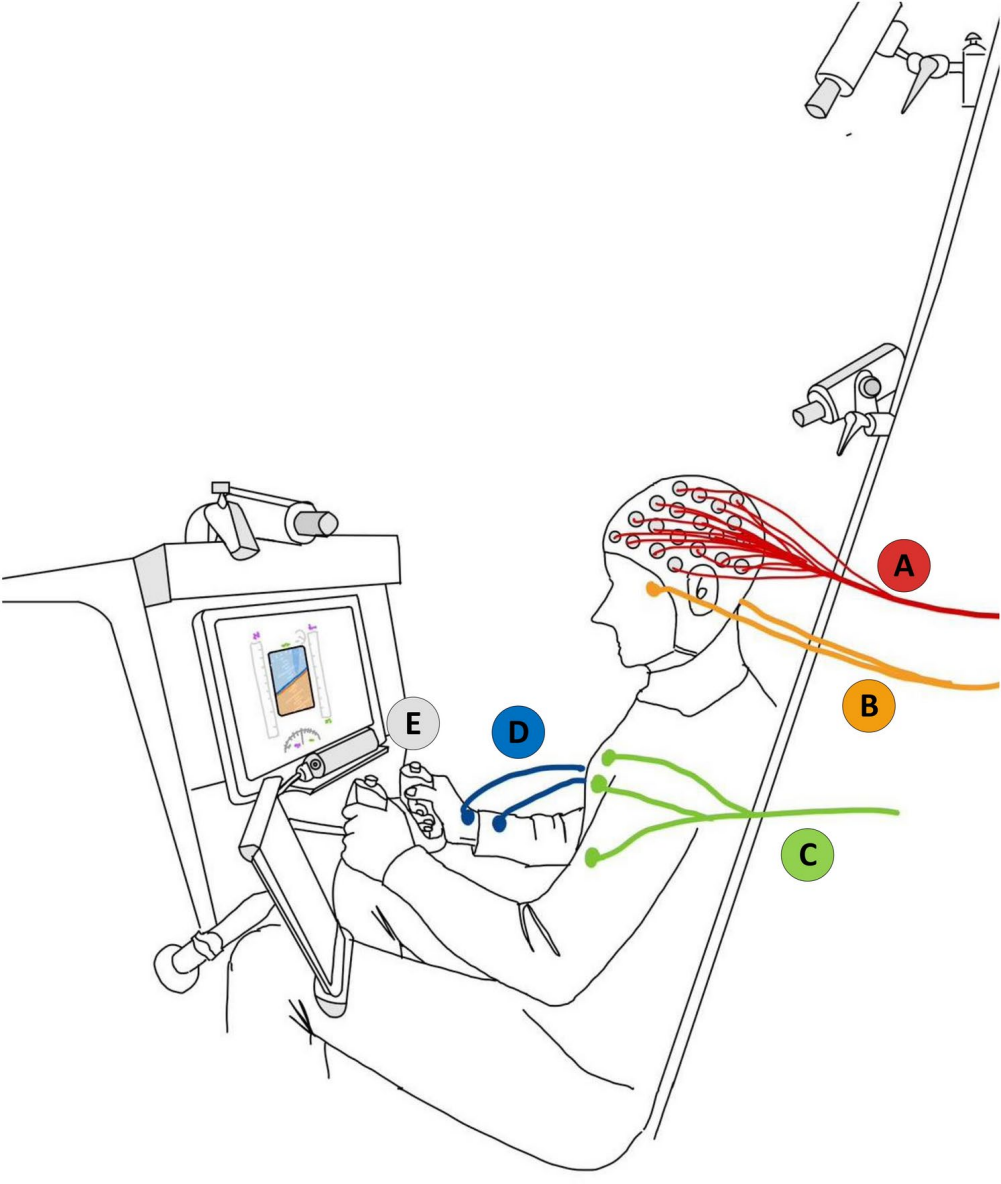
Flight simulator and the experimental settings from the left-side view. The data acquisition includes EEG recordings marked as A, EOG marked as B, ECG marked as C, GSR marked as D, and eye-tracking marked as E.

The experiment was conducted using a custom aircraft flight simulator built by Marinvent Corporation as shown in Figure 1, which was designed to replicate the aircraft dynamics of a Boeing 737. The simulator was run on a computer running the Windows operating system and aircraft dynamics were modeled using XPlane 11 from Laminar Research. The simulator was equipped with a range of controls, including a yoke, rudder pedals, and throttle quadrant. During the experiment, participants did not have access to an out-the-window view but instead relied on a primary flight display (PFD) to monitor the aircraft’s attitude, altitude, heading, climb rate, and speed. Participants used the aircraft yoke to control the aircraft’s pitch and roll, while an autopilot was used to control the throttles (for speed maintenance) and pedals (for turn coordination) aiming to limit the training sessions to basic flight control tasks. As a result, during our pilot training experiments, the task descriptions were designed to request participants’ maneuvers in completing the given tasks by only operating the yoke (Figure 1).

Twenty-four participants aged between 21 and 41 (11 males, 13 females) were recruited to conduct the experiments. Inclusion criteria included being aged between 21 and 45, having normal or corrected-to-normal vision, and no history of neurological or psychiatric disorders. Exclusion criteria included previous pilot training or flight experience, any cognitive impairments, or use of medications that could interfere with cognitive function. These criteria ensured that the participants met the study requirements and reduced potential confounding factors. Each participant received $\$ 100$ CAD compensation, as well as $\$ 80$ CAD for transportation expenses upon completion of experiments. Prior to taking part in the experiment, all participants signed informed consent forms and completed a questionnaire related to the training tasks, and underwent several preparation steps to ensure they had a basic understanding of flight instruments and maneuvers before beginning the pilot training process. Based on their responses, the experimenters interviewed participants for additional information.

In this study, our analysis focuses exclusively on novice pilots with no prior flight skills which aligns with the designing of the experiment protocol and flight tasks. In our study, 29.2$\%$ of the participants reported having some experience with flight simulators, this experience did not translate into actual flight maneuvering skills, as confirmed by our training instructor. The remaining 70.8$\%$ of participants had no prior exposure to flight simulators or flight-related training. Although some participants had interacted with flight simulators before, none possessed the foundational flight skills necessary to be considered anything other than novice for the purposes of this study (Table S11). This distinction is critical for interpreting the results and the impact of previous flight experience, as our findings specifically pertain to individuals without prior flight expertise.

To be more specific, during the preparation they were first required to read a flight briefing presentation in a study room accompanied by one experimenter, to whom they could raise any questions while reading the presentation slides. Then they were asked to stay in the same room to watch four training videos that were prepared by a group of experienced pilot instructors showing how to operate the flight simulator. Afterward, two experimenters brought participants to another room and attached the sensors (EEG, ECG, GSR, EOG) to participants. Once finished, participants were directed to the room equipped with the flight simulator for a familiarization session. During the familiarization session, participants sat in the simulator to practice the basic maneuvers they just learned from the slides and videos. Our test instructor not only showed them how to operate the Yoke as flight control and its effects on pitch and roll but also taught them how to interpret control and performance indications on the PFD, as well as some basic strategies.

Participants took a short break after the familiarization session before the commencement of the pilot training experiment. Once the experiment started, we kept recording participants’ learning behaviors and physiological data until the end of the experiment. The National Research Council’s Integrated Physiological Monitoring (IPM) system^49^ was used to record participants’ physiological data, including Electroencephalogram (EEG), Electrocardiogram (ECG), Galvanic Skin Response (GSR), and Eye tracking (Figure 1). As illustrated in Figure 1, data are captured using: a 64-channel EEG cap marked as A, 2-cable EOG positioned around the eyes marked as B, 3-cable ECG with electrodes placed on the chest (two on the left, one on the right) marked as C, 2-cable GSR with electrodes on the same wrist marked as D, and eye-tracking to monitor gaze behavior marked as E. The aircraft simulator and the IPM system were synchronized using a network time protocol server. An iPad, located on the participant’s left side, was used to present task instructions and collect NASA-TLX ratings, and other questionnaire data (e.g., fatigue ratings) using Qualtrics software. Three cameras, placed at the top, front, and side of the participants, recorded participants’ learning behaviors in the training devices. The EEG data were collected using a 64-channel BioSemi ActiveTwo system placed according to the international 10-20 system at a sampling rate of 2048 Hz. The results from other recorded physiological data were not included in this research. Due to COVID-19 health restrictions, a review of parametric data and video of the sessions was conducted post-experiment for comparative analysis as real-time subjective evaluation by an instructor was not possible. The recorded pilot training process consisted of twenty-two sessions, where the instructions for required maneuvers were displayed on an iPad under the supervision of the Test Director. Participants began with a resting period of two minutes (with eyes open and closed) before the training process commenced. This protocol was approved by both the Concordia Human Research Ethics Committee and the research ethics board of the National Research Council Canada.

**Fig. 2:**
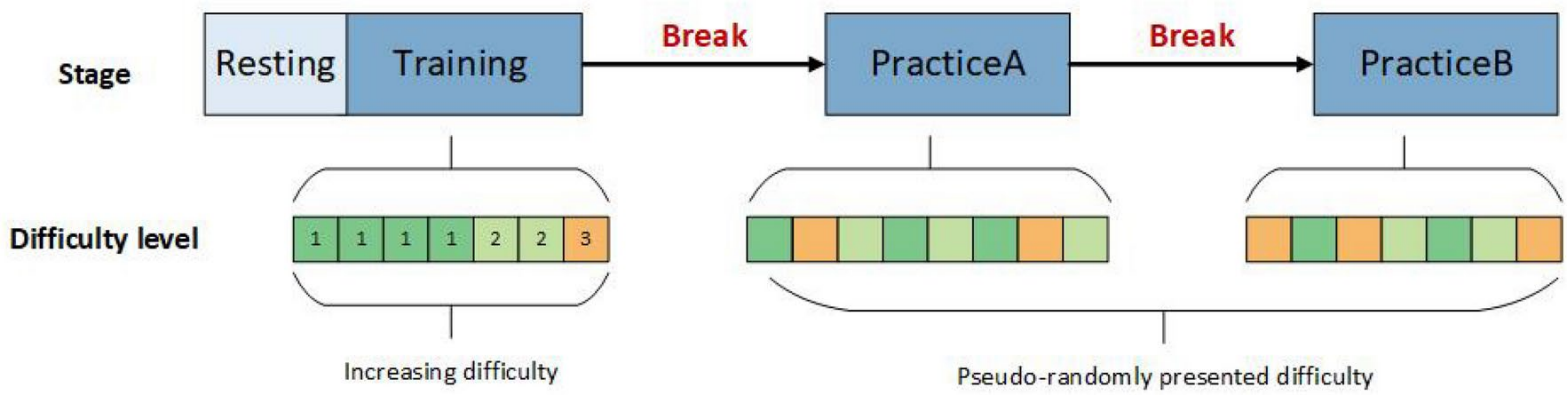
Schematic of the experimental protocol for the tested pilot training process.

Each session followed a similar structure, beginning with a Baseline task for 30 seconds, followed by a Trial task lasting 90 seconds, after which participants completed the NASA-TLX questionnaire to assess their perceived workload. The Baseline tasks required maintaining straight and level flight with constant heading and altitude, while Trial tasks involved performing maneuvers with varying levels of difficulty. Between the Baseline and Trial tasks, participants were given unlimited time to read the task instructions. The twenty-two sessions were divided into three distinct stages (illustrated in Figure 2): Training, Practice A, and Practice B.

- **Training Stage:** Comprised of 7 Baseline and 7 Trial tasks (7 sessions), the difficulty level was progressively increased. The test director provided feedback after each task to participants during this stage, and all participants followed the same sequence of maneuvers, as shown in the “Increasing difficulty” block (green to orange).
- **Practice A stage:** Containing 8 Baseline and 8 Trial tasks (8 sessions), this stage introduced pseudo-randomized task difficulty levels, with no feedback from the instructor. Task difficulty for each Trial task was drawn from three levels (Levels 1, 2, and 3), with one task from each level appearing in every block of three, as shown in the figure.
- **Practice B stage:** Similar to Practice A, this stage included 7 Baseline and 7 Trial tasks (7 sessions), with difficulty levels pseudo-randomly presented. Importantly, a block of three tasks never began with the same level as the last task of the previous block.

### Data pre-processing

The EEG data collected were pre-processed using the EEGLAB toolbox to remove noises^50^. The data were first referenced to mastoids and then filtered between 1 and 40Hz using a zero-phase Hamming windowed-sinc FIR filter to remove artifacts related to low-frequency body movements and focus on the main frequency bands associated with brain activity. Channels that met one or more of the following criteria below were identified as bad channels: 1) the channels that remained flat for more than 5 seconds; 2) channels with a correlation coefficient smaller than 0.8 with their neighboring channels; and 3) channels with amplitudes greater than 3 standard deviations from the mean. In addition, the number of bad channels identified under each task can be found in Supplementary Table S2. For artifact removal, we applied an automatic artifact removal process via MARA, the multiple artifact rejection algorithm^51^, to provide a consistent, objective approach to artifact rejection across all participants and datasets, reducing the variability that can arise from manual operations. MARA was applied to identify and remove the IC components^52^ having more than 40$\%$ chance to be labeled as artifacts (eye-blink, eye-movement, muscle-generated, and other artifacts). More details about the remaining independent components could be found in Supplementary Table S3. Moreover, the signals were segmented in 2-second epochs for detecting bad segments and bad local channels within each segment^53^.

Bad local channels in each segment were detected using FASTER^54^ criteria (variance, median gradient, amplitude range, and deviation from mean amplitude) when one or more Z scores of four criteria were greater than 3 standard deviations from the mean, which were interpolated thereafter using spherical splines. Afterward, bad segments were identified and rejected when one or more criteria were satisfied: a channel’ amplitude was higher than $\pm 100 \, \mu \text {V}$; the single electrode probability across segments or the electrode group probability within segments was greater than 3 standard deviations from the mean. Finally, the isolated bad global channels were interpolated using spherical splines. The cleaned EEG signals were re-referenced to the average reference and were downsampled to 250 Hz.

### Spectral analysis

Spectral features were computed and compared in both STAGE comparisons and TASK comparisons to investigate the time-course changes and to align our analysis with other findings reported in the literature. The power spectral density (PSD) was first computed by applying the Welch periodogram method with $50\%$ overlapping Hamming windows of a length of 2 seconds to the pre-processed EEG data. Afterward, the theta band (4-7.5 Hz) powers^55^ were computed from the PSD for each Trial task and Baseline task separately within the tested training process (22 Trial tasks, and 22 Baseline tasks). Under each task, the band powers at 64 EEG channels were then grouped into five cortical areas (frontal, central, temporal, parietal, and occipital)^56,57^ for including AREA comparisons in repeated measures ANOVA.

### EEG microstate analysis

We computed seven microstate classes (A, B, C, D, E, F, G) following the approach proposed by Pascual-Marqui et al^58^. For each task at each stage, we computed the Global Field Power (GFP) of the pre-processed EEG data and sent only the EEG data at the computed GFP peaks to the modified k-means algorithm with the cost function defined in Eq. (1). We repeated this process 100 times to select the optimal microstate classes based on cross-validation, as defined in Eq. (2), from the microstate classes computed in each repetition.

1 $$\begin{aligned} F = \frac{1}{N_T(N_S-1)}\sum _{t=1}^{N_T}||V_t - \sum _{k=1}^{N_K}a_{kt}\Gamma _k ||^2, \end{aligned}$$

2 $$\begin{aligned} CV = \frac{\sum _{t=1}^{N_T} (V_t^\prime\cdot V_t - (V_t^\prime\cdot \Gamma _k)^2)}{N_T(N_S-1)}\cdot (\frac{N_S-1}{N_S-1-N_K})^2, \end{aligned}$$

where $N_T$ is the sample length, $N_S$ is the number of electrodes, $V_t$ is a $N_S \times 1$ vector consisting of the electric potential at time point *t*, $N_K$ is the number of microstate classes, $\Gamma _k$ is a normalized $N_S \times 1$ vector representing the k-th microstate class, $a_{kt}$ is the intensity of the k-th microstate class at the time point t.

Next, we applied a full permutation procedure to compute group-wise microstate classes from the microstate classes obtained for each participant’s single task, which were referred to as global microstate classes. We represented the pre-processed EEG data in the time domain by assigning one of the global microstate classes to each time point, using the highest spatial correlation as the criterion for microstate class assignment and ignoring the polarity of the microstate classes. Moreover, no smoothing parameters were applied in our analysis to avoid any modification of the temporal dynamics of the generated microstate sequences.

Thereafter, three microstate parameters, namely duration, occurrence, and coverage, were computed for the 44 microstate sequences (22 sequences for Baseline tasks, 22 sequences for Trial tasks) generated for each participant’s different tasks during varied stages. These three parameters were used to describe how long a microstate could remain stable, how many times a microstate appeared per second, and the fraction of the total analysis time covered by a microstate, respectively.

Moreover, a finite estimate of the entropy rate^59^ and Hurst exponent estimated by detrended fluctuation analysis (DFA)^60^ were applied to measure the temporal dependencies of different microstates. In particular, the entropy rate was used to measure short-range temporal dependencies, while the Hurst exponent was used to measure long-range temporal dependencies^57^. The generated microstate sequences were mapped into the metric space $S_0=\{-1, +1\}$ before applying DFA for Hurst exponent computation. A total of 35 partitions were obtained for the seven microstate classes, and the Hurst exponent was computed for the mapped sequences generated under each partition. Afterward, the Hurst exponents averaged across 35 partitions were used to describe the long-range temporal dependencies.

### Statistical analysis

The EEG spectral power changes in theta band were analyzed using a 2 (TASK)$\times 3$ (STAGE)$\times 5$ (AREA) repeated measures ANOVA with three within-subject factors: TASK (Baseline and Trial), STAGE (Training, PracticeA, and PracticeB), and AREA (Frontal, Central, Temporal, Parietal, and Occipital). Greenhouse-Geisser correction was applied in the case of sphericity violations. Post hoc paired t-test was conducted between TASK and between STAGE at each tested AREA with Bonferroni correction for multiple comparisons.

For each computed EEG microstate parameter (coverage, occurrence, and duration), a 2 (TASK) $\times 3$ (STAGE)$\times 7$ (CLASS) repeated measures ANOVA was applied to analyze the effects of different factors. The three within-subject factors were TASK (Baseline and Trial), STAGE (Training, PracticeA, and PracticeB), and CLASS (A - G). Greenhouse-Geisser correction was applied in the case of sphericity violations. Post hoc paired t-test was conducted between TASK and between STAGE at each microstate CLASS with Bonferroni correction for multiple comparisons.

The temporal dependencies measured by entropy rate and Hurst exponent were analyzed using a 2 (TASK) $\times 3$ (STAGE) repeated measures ANOVA. The two within-subject factors were TASK (Baseline and Trial) and STAGE (Training, PracticeA, and PracticeB). Greenhouse-Geisser correction was applied in the case of sphericity violations. Post hoc paired t-test was conducted between TASK and between STAGE with Bonferroni correction for multiple comparisons.

## Results

### Subjective evaluation results

The subjective evaluation of pilot trainees’ aircraft control performance was conducted using a combination of qualitative and quantitative assessment. The evaluation process involved a comprehensive assessment of the training trials across three distinct aspects encompassing the quality of the dataset, a quantitative evaluation of performance in maneuvering the simulated aircraft under specific tasks, and a descriptive analysis of the actions undertaken by the trainees. To ensure an appropriate grading scale, a panel of two instructors collaborated to establish the levels of evaluation tailored to the expected performance of ab-initio candidates for each parameter. To minimize variability in assessments, a single qualified instructor was designated to evaluate the pilot trainees’ control skills of the simulated aircraft.

**Fig. 3:**
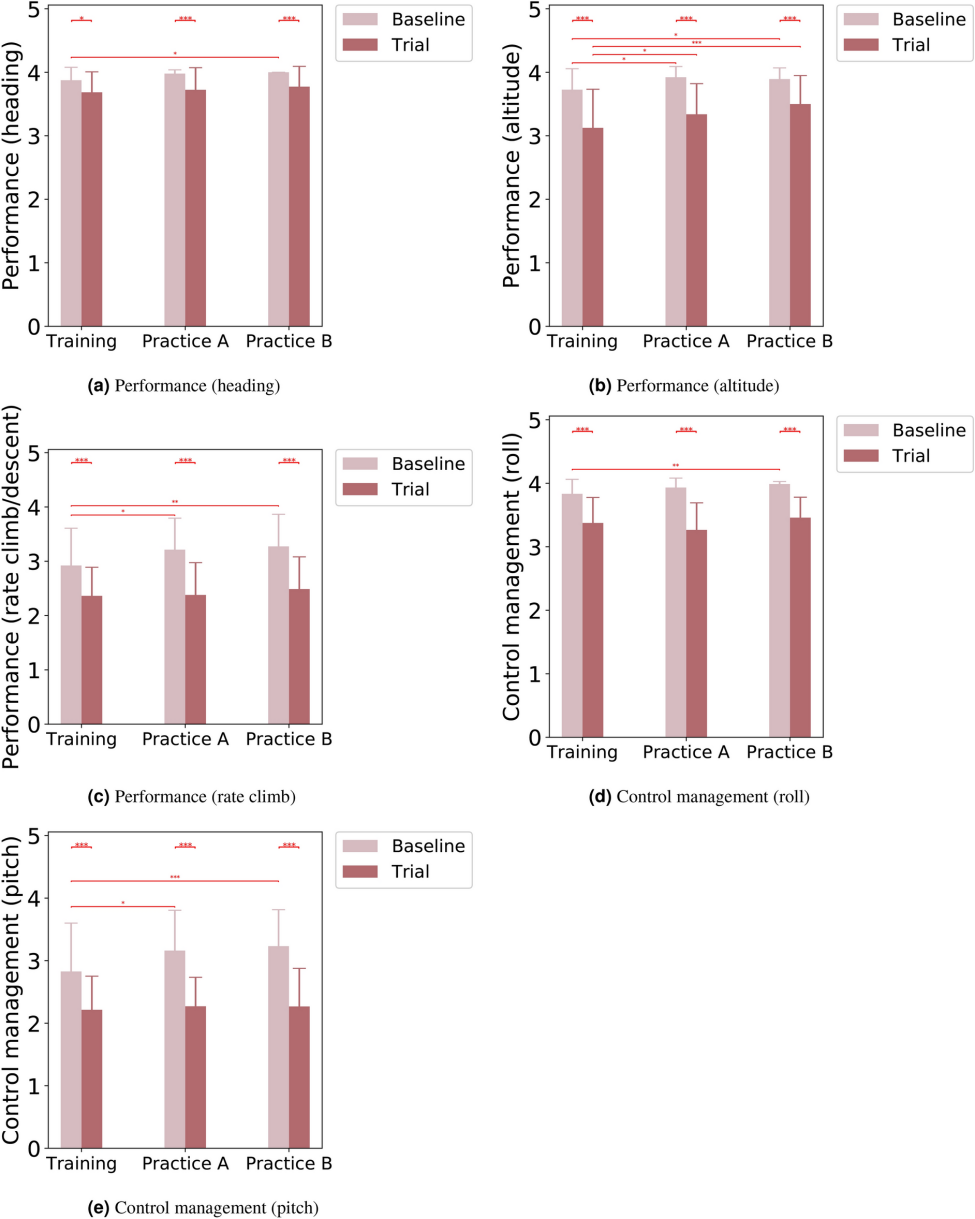
Quantitative assessment on each dimension with P-values for TASK and STAGE comparisons annotated by $$^{*}(p \le 0.050)$$, $$^{**}(p \le 0.010)$$, $$^{***}(p \le 0.005)$$.

**Table 1:** Averaged results for each performance evaluation dimension under Baseline and Trial tasks.

Mean (SE)	Training		PracticeA		PracticeB	
	Baseline	Trial	Baseline	Trial	Baseline	Trial
P-H	3.88 (0.042)	3.68 (0.066)	3.98 (0.012)	3.72 (0.000)	4.00 (0.000)	3.77 (0.065)
P-A	3.73 (0.067)	3.13 (0.124)	3.92 (0.034)	3.34 (0.098)	3.89 (0.036)	3.50 (0.091)
P-R	2.92 (0.140)	2.36 (0.107)	3.21 (0.118)	2.38 (0.121)	3.27 (0.121)	2.49 (0.121)
C-R	3.83 (0.047)	3.38 (0.082)	3.93 (0.030)	3.27 (0.087)	3.99 (0.008)	3.46 (0.066)
C-P	2.83 (0.158)	2.21 (0.110)	3.16 (0.132)	2.27 (0.095)	3.23 (0.119)	2.27 (0.124)

Among the three aspects analyzed, the quality of the dataset is inherently reflected in the results obtained from the quantitative assessment of the trainees’ aircraft control performance. However, the descriptive analysis provides more information that can contribute to a more comprehensive understanding of the factors influencing trainees’ control performance, whether positive or negative. For this research, the focus was primarily on the quantitative assessment aspect, which not only effectively reflects pilot trainees’ proficiency in controlling the simulated aircraft throughout the training process, but also considers the control behavior recorded by the simulator into consideration.

The quantitative assessment was composed of five dimensions in total including three types of performance (heading, altitude, and rate climb/descent) and two types of control management evaluations (roll and pitch), which were denoted as P-H, P-A, P-R, C-R, and C-P in Table 1 and Supplementary Table S4. The grading process was conducted post-experiment, involving the conversion of all trial and baseline simulator-based records into charted plots, which were then collated and presented to the evaluator for assessment. These records encompassed critical flight metrics such as pitch, altitude, rate of climb/descent, bank, heading, and the candidate’s flight control inputs in both pitch and roll. Each task was accompanied by a task description, and the evaluator utilized a quantitative grading method to assess each evaluation dimension. All performance parameters, including pitch and bank control, were graded on a scale ranging from 1 to 4, with 1 representing the lowest accuracy and 4 denoting the highest. A grading matrix was employed to establish a range of performance accuracy for each dimension. Given the dynamic nature of the maneuvers, the evaluator projected an ideal target profile variation, which was then compared to the candidate’s actual profile. This approach allowed for some flexibility in the candidate’s time strategy while ensuring adherence to task requirements and grading criteria.

The quantitative assessment decreased significantly from Baseline tasks to Trial tasks for all five evaluated dimensions throughout the training process (including Training, PracticeA, and PracticeB stages) as shown in Figure 3a-3e. The average evaluation results are listed in Table 1 and the related p-values for paired TASK comparisons can be found in Supplementary Table S4. For STAGE comparisons on Baseline tasks, significant increases from Training to PracticeA stage were observed under three evaluated dimensions, namely performance (altitude) (Figure 3a), performance (rate climb/descent) (Figure 3c), and control management (pitch) (Figure 3e). Such significant increases were also observed from Training to PracticeB stages for all five evaluated dimensions (Figure 3a-3e) when trainees were conducting Baseline tasks. For STAGE comparisons on Trial tasks, performance (altitude) was the only dimension that showed significant increases from Training to PracticeA as well as from Training to PracticeB stage (Figure 3b). The related p-values for STAGE comparisons with Bonferroni correction are listed in Supplementary Table S4.

### EEG spectral power results

**Fig. 4:**
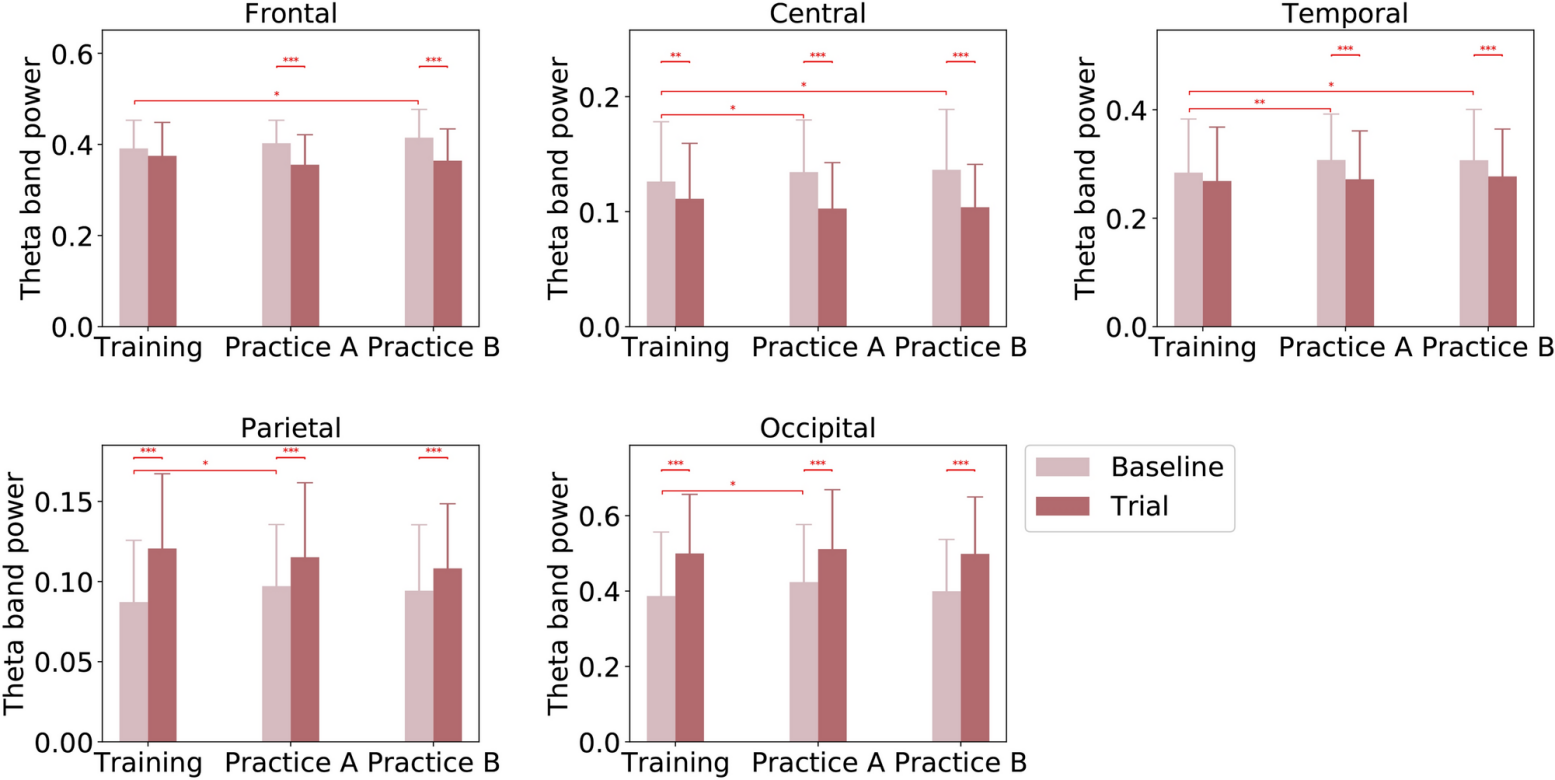
EEG theta band power for Baseline tasks and Trial tasks during Training, PracticeA, and PracticeB. P-values for TASK and STAGE comparisons are annotated by $$^{*}(p \le 0.050)$$, $$^{**}(p \le 0.010)$$, $$^{***}(p \le 0.005)$$.

In the theta band, the $2\times 3\times 5$ repeated measures ANOVA revealed two significant main effects of TASK ($F (1,23)=13.110$, $p =0.001$, $\eta ^2=0.368$) and AREA ($F (4,92)=34.337$, $p =0.000$, $\eta ^2=0.600$), as well as one significant interaction effect of TASK $\times$ AREA ($F (4,92)=15.604$, $p =0.000$, $\eta ^2=0.420$). Figure 4 presents the comparison results on TASK and STAGE on each brain area. The p-values for pairwise comparisons of theta spectral power with Bonferroni correction on each area are listed in Supplementary Table S5, where $B-$ and $T-$ represent Baseline tasks and Trial tasks respectively, while *S*1, *S*2, and *S*3 correspond to Training, PracticeA, and PracticeB stages respectively. As shown in Figure 4, trainees’ theta spectral power decreased significantly from Baseline tasks to Trial tasks over frontal sites under PracticeA stage and PracticeB stage, over central sites for all three stages (Training, PracticeA, and PracticeB), and over temporal sites under PracticeA stage and PracticeB stage. In the meantime, significant increases in theta band powers were observed from Baseline tasks to Trial tasks over parietal and occipital sites under the Training stage, PracticeA stage, and PracticeB stage. As for STAGE comparison on Baseline tasks, the spectral power results in theta band showed significant increases from the Training stage to PracticeA stage over central, temporal, parietal, and occipital sites, as well as from the Training stage to PracticeB stage over frontal, central, and temporal sites. However, no significant changes were observed between different stages while the trainees were conducting Trial tasks over the five brain areas.

### EEG microstate analysis

**Fig. 5:**
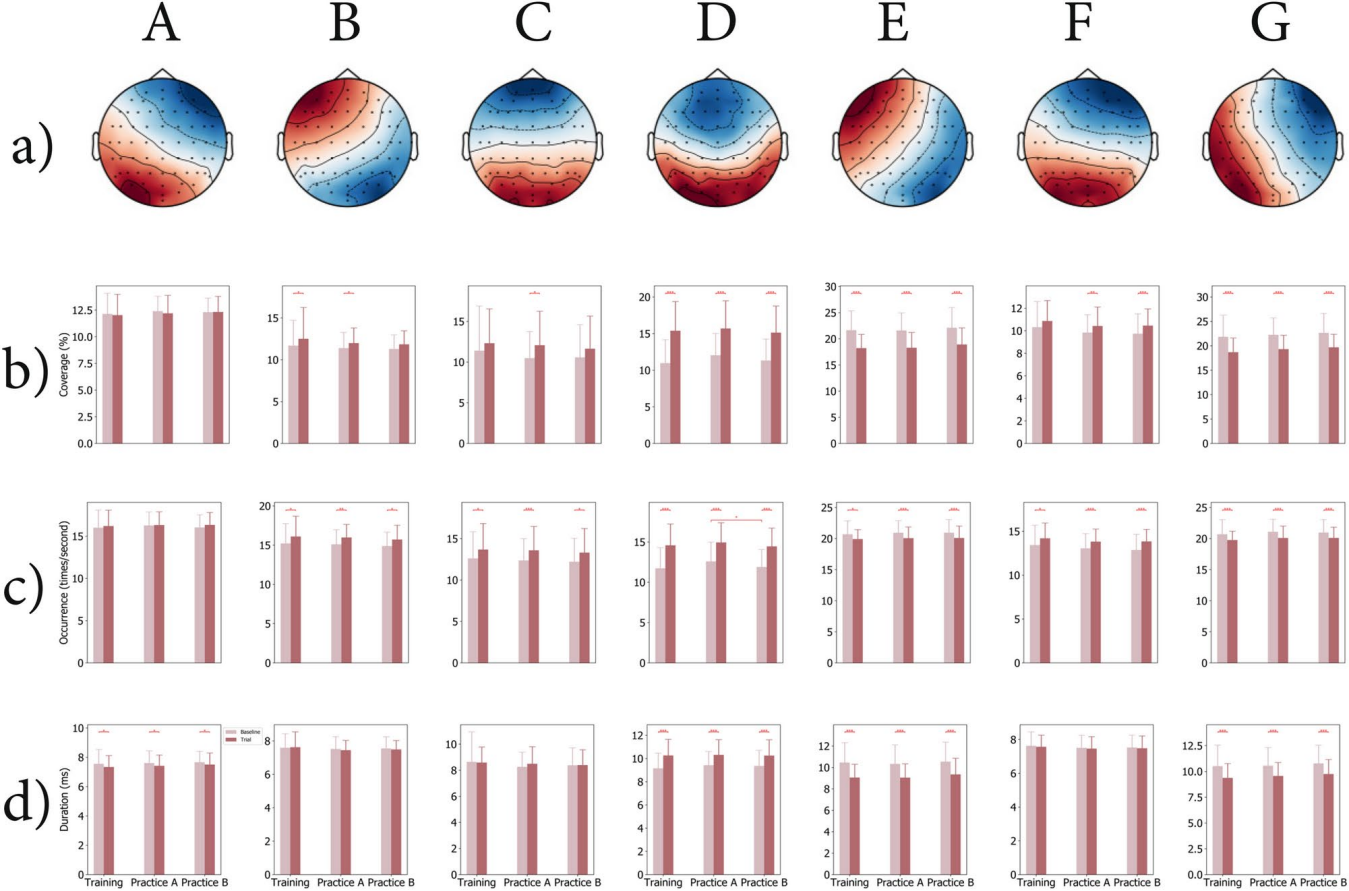
EEG microstate analysis across Training, Practice A, and Practice B stages for both Baseline and Trial tasks. a) Spatial configuration of the seven global EEG microstate classes across the different task types and stages. b) Microstate coverage of each microstate class during Baseline and Trial tasks at each stage. c) Microstate occurrence of each microstate class during Baseline and Trial tasks at each stage. d) Microstate duration of each microstate class during Baseline and Trial tasks at each stage. P-values for TASK and STAGE comparisons in b)-d) are annotated by $$^{*}(p \le 0.050)$$, $$^{**}(p \le 0.010)$$, $$^{***}(p \le 0.005)$$.

Figure 5-a) shows the topographic maps of seven global microstate classes across TASK and across STAGE, and the microstate classes under each task type (Baseline or Trial) during different stages (Training, PracticeA, and PracticeB) could be found in Supplementary Fig. S3. The seven microstate classes were labeled as A, B, C, D, E, F, and G according to Custo et al. (2017)^61^, Michel and Koenig (2018)^40^, and Jia et al. (2021)^57^. For Baseline tasks, the seven microstate classes explained $$66.685\%$$$$(SE=0.347)$$ of the global variance of the original EEG topographies corresponding to peaks of GFP for the Training stage, $$67.628\%$$$$(SE=0.294)$$ for PracticeA stage, $$68.749\%$$$$(SE=0.312)$$ for PracticeB stage. For Trial tasks, the seven microstate classes explained $$67.127\%$$$$(SE=0.348)$$ of the global variance of the original EEG topographies corresponding to peaks of GFP for the Training stage, $$67.894\%$$$$(SE=0.264)$$ for PracticeA stage, $$68.342\%$$$$(SE=0.301)$$ for PracticeB stage.

The computed EEG microstate parameters include coverage, occurrence, and duration. For microstate coverage analysis, the $2\times 3\times 7$ repeated measures ANOVA revealed one significant main effect of CLASS ($F (6,138)=52.261$, $p =0.000$, $\eta ^2=0.694$), as well as one significant interaction effect of TASK $\times$ CLASS ($F (6,138)=37.493$, $p =0.000$, $\eta ^2=0.620$). Figure 5-b) presents the coverage comparison results on TASK and STAGE for each of the seven EEG microstate classes, while the p-values for pairwise comparisons with Bonferroni correction on each microstate class are listed in Supplementary Table S6. For TASK comparison, significant increases in the coverage of microstate B from Baseline tasks to Trial tasks were observed for Training and PracticeA stages; the significant changes from Baseline to Trial tasks were also observed for the coverage of microstate C for PracticeA (increasing); the coverage of microstate D for Training (increasing), PracticeA (increasing), and PracticeB (increasing); the coverage of microstate E for Training (decreasing), PracticeA (decreasing), and PracticeB (decreasing); the coverage of microstate F for PracticeA (increasing) and PracticeB (increasing); the coverage of microstate G for Training (decreasing), PracticeA (decreasing), and PracticeB (decreasing). The results on EEG microstate coverage showed no significant difference in STAGE comparisons in all of the seven microstate classes.

In addition, the $2\times 3\times 7$ repeated measures ANOVA on EEG microstate occurrence revealed two significant main effects of TASK ($F (1,23)=17.349$, $p =0.000$, $\eta ^2=0.430$) and CLASS ($F (6,138)=91.163$, $p =0.000$, $\eta ^2=0.799$), as well as one significant interaction effect of TASK $\times$ CLASS ($F (6,138)=32.424$, $p =0.000$, $\eta ^2=0.585$). Figure 5-c) presents the occurrence comparison results on TASK and STAGE for each of the seven EEG microstate classes, while the p-values for pairwise comparisons with Bonferroni correction on each microstate class are listed in Supplementary Table S7. For TASK comparison, significant differences between Baseline tasks and Trial tasks were observed for all three stages (Training, PracticeA, and PracticeB) in the occurrence of microstate B, C, D, E, F, and G despite the polarity of the changes. To be more precise, significant increases were observed in the occurrence of microstate B, C, D, and F during Training, PracticeA, and PracticeB, whereas significant decreases were observed in the occurrence of microstate E and G during Training, PracticeA, and PracticeB. No significant difference was observed in the occurrence of microstate A under TASK comparisons. For STAGE comparison, the occurrence of microstate D decreased significantly from PracticeA stage to PracticeB stage for Baseline tasks, whereas none of the seven microstate classes showed any significant occurrence difference in STAGE comparisons for Trial tasks.

Moreover, the $2\times 3\times 7$ repeated measures ANOVA on EEG microstate duration revealed two significant main effect of TASK ($F (1,23)=12.140$, $p =0.002$, $\eta ^2=0.345$) and CLASS ($F (6,138)=35.832$, $p =0.000$, $\eta ^2=0.609$), as well as one significant interaction effect of TASK $\times$ CLASS ($F (6,138)=31.065$, $p =0.000$, $\eta ^2=0.575$). Figure 5-d) presents the duration comparison results on TASK and STAGE for each of the seven EEG microstate classes, while the p-values for pairwise comparisons with Bonferroni correction on each microstate class are listed in Supplementary Table S8. For TASK comparison, significant increases in the duration of microstate D from Baseline tasks to Trial tasks were observed for Training, PracticeA, and PracticeB stages, whereas the duration of microstate A, E, and G decreased significantly from Baseline to Trial tasks for Training, PracticeA, and PracticeB stages. No significant differences between Baseline tasks and Trial tasks were observed in the duration of microstates B, C, and F across different stages. In the meantime, the results on EEG microstate duration showed no significant difference in STAGE comparisons for all seven microstate classes for both task types (Baseline and Trial).

### Temporal dependencies of EEG microstates

**Fig. 6:**
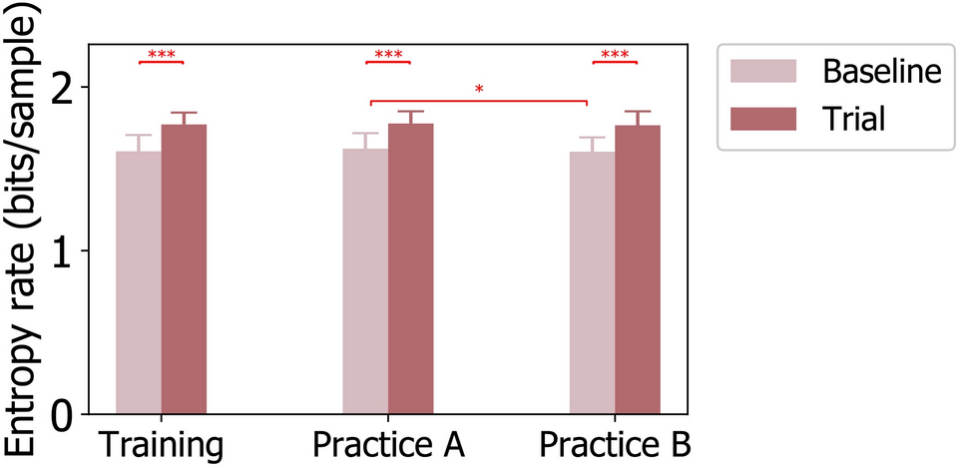
Entropy rate of microstate sequences for Baseline and Trial tasks during Training, PracticeA, and PracticeB stages. P-values for TASK and STAGE comparisons are annotated by $$^{*}(p \le 0.050)$$, $$^{**}(p \le 0.010)$$, $$^{***}(p \le 0.005)$$.

For Baseline tasks, the finite entropy rate was 1.607 *bits*/*sample*$(SE=0.020)$ during Training, 1.623 *bits*/*sample*$(SE=0.019)$ during PracticeA, and 1.604 *bits*/*sample*$(SE=0.018)$ during PracticeB. For Trial tasks, the finite entropy rate was 1.771 *bits*/*sample*$(SE=0.015)$ during Training, 1.776 *bits*/*sample*$(SE=0.015)$ during PracticeA, and 1.766 *bits*/*sample*$(SE=0.017)$ during PracticeB. The $2\times 3$ repeated measures ANOVA revealed one significant main effect of TASK ($F (1,23)=291.901$, $p =0.000$, $\eta ^2=0.927$). Figure 6 presents the entropy rate results for TASK and STAGE comparisons, whereas the p-values for pairwise comparisons with Bonferroni correction are listed in Supplementary Table S9. For TASK comparison, the average entropy rate of microstate sequences increased significantly from Baseline tasks to Trial tasks for all of the three tested stages including Training, PracticeA, and PracticeB. As for STAGE comparisons, the entropy results showed significant decreases from PracticeA to PractiveB stage, whereas no significant difference in STAGE comparisons was observed for Trial tasks.

**Fig. 7:**
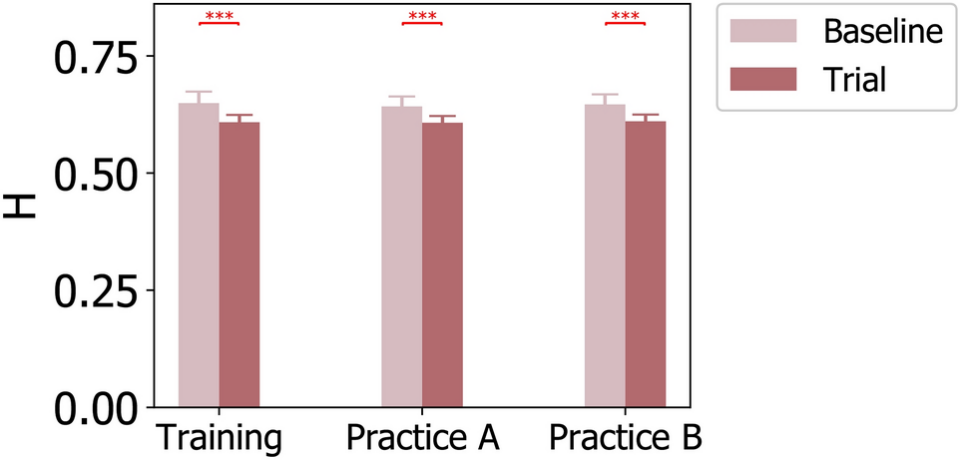
Hurst exponent of microstate sequences averaged across 35 partitions for Baseline and Trial tasks during Training, PracticeA, and PracticeB stages. P-values for TASK and STAGE comparisons are annotated by $$^{*}(p \le 0.050)$$, $$^{**}(p \le 0.010)$$, $$^{***}(p \le 0.005)$$.

Moreover, the Hurst exponent averaged across 35 partitions was 0.649 $(SE=0.005)$ for Training, 0.642 $(SE=0.004)$ for PracticeA, and 0.647 $(SE=0.004)$ for PracticeB under Baseline tasks. For Trial tasks, the finite entropy rate was 0.608 $(SE=0.003)$ for Training, 0.607 $(SE=0.00.)$ for PracticeA, and 0.611 $(SE=0.003)$ for PracticeB. The $2\times 3$ repeated measures ANOVA revealed one significant main effect of TASK ($F (1,23)=221.604$, $p =0.000$, $\eta ^2=0.906$). Figure 7 presents the average Hurst exponent for TASK and STAGE comparisons, whereas the p-values for pairwise comparisons with Bonferroni correction are listed in Supplementary Table S10. For TASK comparison, significant decreases were observed in the averaged Hurst exponent of microstate sequences from Baseline tasks to Trial tasks for all three tested stages including Training, PracticeA, and PracticeB. However, no significant difference was observed in the Hurst exponent results on STAGE comparisons for both Baseline and Trial tasks.

## Discussion

### Trainees’ cognitive control under varied task demands

In accordance with our experimental protocol, the distinction between Baseline tasks and Trial tasks lies primarily in the novelty and task demands. Under Baseline tasks, trainees engage in repetitive tasks, whereas Trial tasks present varied challenges from one instance to another. This variation necessitates trainees to employ different cognitive processes when encountering familiar versus unfamiliar materials. Moreover, Trial tasks entail larger task loads and difficulty compared to Baseline tasks, further amplifying the demands and uncertainties faced by trainees under Trial tasks. While each Baseline task repeated the same task description and lasted for 30 seconds, Trial tasks lasted for 90 seconds and varied in the requested maneuvers. In general, the maneuvers required under Trial tasks were more difficult than those under Baseline tasks, and the instructions for each of the twenty-two Trial tasks differed from one another. To align our research with the reported effect of task demands, difficulty, and uncertainty on cognitive control in the literature^17,62–67^, we conducted paired comparisons between the two types of tasks: Baseline tasks and Trial tasks.

The paired comparisons between the Trial and Baseline tasks revealed that trainees’ cognitive control decreased as they encountered tasks with higher difficulty levels and increased uncertainty. The supporting evidence includes a decrease in theta spectral power, an increase in the parameters of microstate C, an increased entropy rate, and a decreased Hurst exponent under Trial tasks compared to Baseline tasks. Significant decreases in EEG spectral power in the theta band were observed from Baseline tasks to Trial tasks during the Training stage over central sites and during both PracticeA and PracticeB stages over frontal, central, and temporal sites. In the literature, increased theta power over frontal sites is considered a reliable indicator of working memory and cognitive control^27,68^, which could be associated with enhanced cognitive control during the encoding and retrieval of information from working memory^69,70^. Overall, our analysis indicates a decrease in trainees’ cognitive control under increased task demands and uncertainty during the Trial tasks, while significant increases in theta power over parietal and occipital sites may reflect increased visual loads.

Among the seven computed EEG microstate states, microstate C was discussed here due to its close relationship with cognitive control. Researchers have reported both positive and negative correlations of microstate class C with cognitive control mechanisms as reviewed in (Michel and Koenig, 2018)^40^. As a result, TASK comparisons were conducted on the three parameters of microstate C, namely coverage, occurrence, and duration, and the results indicated increased microstate C under Trial tasks. To be more precise, the coverage of microstate C increased from Baseline tasks to Trial tasks during the Training stage (non-significant), PracticeA stage (significant), and PracticeB stage (non-significant) as shown in Figure 5-b). The occurrence of microstate C significantly increased from Baseline tasks to Trial tasks throughout the pilot training process (Training, PracticeA, and PracticeB) (Figure 5-c)), whereas the duration of microstate showed non-significant decreases under the Training stage and non-significant increases under PracticeA and PracticeB stages (Figure 5-d)). Combining the observations on microstate C and those indicated by other EEG quantities, the increased microstate C under Trial tasks indicated that trainees’ cognitive control decreased under larger task demands with increased uncertainty. Therefore, our results supported the notion that microstate class C is negatively correlated to task demands, which was consistent with the studies suggesting microstate C’s role in reflecting activities in the default mode network (DMN)^42–44^.

The entropy rate computed from EEG microstate sequences described how random the sequences were, as it described how random or free the brain was in choosing the next network configuration^57^. The more random a microstate sequence was, the larger the computed entropy rate became. On the contrary, the more organized a sequence was, the smaller the entropy rate could be. In other words, a decreased entropy rate would be associated with a higher cognitive control as both of them indicated a more organized microstate sequence in our analysis. Our paired TASK comparisons showed that the entropy rate significantly increased from Baseline tasks to Trial tasks across the three stages (Training, PracticeA, and PracticeB) within a pilot training process as shown in Figure 6. Given the negative correlation between entropy rate and cognitive control, our analysis of the entropy rate of microstate sequences revealed that trainees’ cognitive control was lower when they were confronted with increased task demands and increased uncertainty under Trial tasks, which was consistent with the changing trend indicated by theta band power and the parameters of microstate C.

Hurst exponent, as an indicator of long-range dependency, reflected the long-range correlation in the EEG microstate sequences. The larger the Hurst exponent was, the more temporally correlated the microstates were, whereas the Hurst exponent at 0.5 represented a completely uncorrelated microstate sequence. From this standpoint, increases in Hurst exponent may indicate a higher cognitive control, both of which are associated with a more predictable microstate sequence. According to our paired TASK comparison results, significant decreases in the computed average Hurst exponent were observed from Baseline to Trial tasks throughout the pilot training process (Training, PracticeA, and PracticeB) as shown in Figure 7. Considering the aforementioned positive correlation between Hurst exponent and cognitive control, our results suggest that trainees had lower cognitive control when faced with increased task demands and uncertainty under Trial tasks. These observations were consistent with those indicated by theta spectral power, the parameters of microstate C, and the entropy rate.

In summary, our findings from the TASK comparisons indicate that trainees had reduced cognitive control when facing higher task demands and increased uncertainty in completing new tasks under the Trial condition. The quantitative performance evaluation results also suggest that trainees were less skilled and experienced more difficulty in completing the Trial tasks compared to the Baseline tasks. Specifically, the significant decreases in each evaluated dimension throughout the training process (Training, PracticeA, PracticeB) complemented the EEG features in indicating that individuals may experience lower cognitive control during tasks that are challenging for them. Our analysis of cognitive control was consistent with the negative correlation between cognitive control and uncertainty reported in previous studies^57,62,63^. For example, our observations of the diminished cognitive control under increased task difficulty are consistent with Riddle et al.’s work^65^ as evidenced by the changes in microstate parameters and spectral features like reduced theta power during the high-demand flight tasks (Trial tasks). The findings from the paper^65^ highlight the neural mechanisms that underlie hierarchical cognitive control in the presence of varying levels of task difficulty. The decreased cognitive control observed in the current study may be partly due to the participants’ challenges in adapting to these more complex task demands, which is similar to how the increase in task complexity in the study^65^ led to distinct changes in neural oscillations. This connection between theta power and cognitive load also aligns with our interpretation of the cognitive control dynamics under increased uncertainty and task demands. Thus, the consistency across the applied EEG quantities and existing literature confirms the reliability of our analysis regarding cognitive control.

### Trainees’ cognitive control under different pilot training stages

The STAGE comparisons on Baseline tasks demonstrated either significant or non-significant increases in theta band power from the Training stage to the PracticeA and PracticeB stages. Notably, there were significant increases in theta band power from the Training stage to the PracticeA stage over central, temporal, parietal, and occipital sites, as well as non-significant increases over frontal sites. Similarly, significant theta increases were observed from the Training stage to the PracticeB stage over frontal, central, and temporal sites, as well as non-significant theta increases over parietal and occipital sites. Furthermore, there were no significant changes in theta spectral powers from the PracticeA stage to the PracticeB stage. According to the literature, increased theta power over the frontal sites has been viewed as a reliable index of working memory and cognitive control^27,68^, which could be related to a higher cognitive control in encoding and retrieval of information from working memory^69,70^. In the same vein, our EEG spectral analysis in the theta band showed that the Training stage was associated with the lowest cognitive control compared to the other two stages (PracticeA and PracticeB), indicating improvements in trainees’ cognitive control from the Training stage to the two practice stages as the training continues.

Regarding the parameters of microstate C, no significant changes were observed in the STAGE comparisons for all three microstate parameters: coverage, occurrence, and duration. When looking at the non-significant changes, the mean coverage of microstate C showed a non-significant decrease from 11.403 in Training to 10.480 in PracticeA, followed by a non-significant increase to 10.582 in PracticeB. Similarly, the mean occurrence of microstate C exhibited a non-significant decrease from 12.600 in Training to 12.366 in PracticeA, and then a non-significant decrease to 12.212 in PracticeB. The mean duration of microstate C also showed a non-significant decrease from 8.635 in Training to 8.258 in PracticeA, and then a non-significant increase to 8.376 in PracticeB. Considering the negative correlation between microstate C and cognitive control, we did not find significant changes in trainees’ cognitive control in STAGE comparisons of those parameters under Baseline tasks due to the lack of significant changes and consistency in the observed trends.

The comparisons of entropy rates between different pilot training stages showed a non-significant increase from the Training stage to the PracticeA stage, followed by a significant decrease from PracticeA to PracticeB stage, and a non-significant decrease from the Training stage to the PracticeB stage under Baseline tasks (Figure 6). To be more precise, the entropy rate increased non-significantly from 1.607 in the Training stage to 1.623 in the PracticeA stage, then decreased significantly from 1.623 in PracticeA to 1.604 in PracticeB under Baseline tasks. Considering the known negative relationship between entropy rate and cognitive control, our STAGE comparisons on entropy rates indicated that pilot trainees’ cognitive control improved from the PracticeA stage to the PracticeB stage which could be a result of repetitive practices.

Furthermore, the comparisons of the Hurst exponent between different stages showed no significant changes even when task demands were held constant under Baseline tasks. That is, non-significant decreases were observed from the Training stage to PracticeA stage and non-significant increases were found from PracticeA stage to PracticeB stage under unvaried task demands (Figure 7), which seemed to indicate a U-shaped changing trend in the average Hurst exponent. To be more precise, the Hurst exponent decreased non-significantly from 0.649 under the Training stage to 0.642 under PracticeA stage, and then increased non-significantly to 0.647 under PracticeB stage. Therefore, the comparisons of the Hurst exponent did not indicate any significant changes in pilot trainees’ cognitive control along the pilot training process, even though there were some non-significant changes that may indicate slight changes in trainees’ control ability.

Moreover, the non-significant changes observed in the tested two temporal dependency features (entropy rate and Hurst exponent) indicated a U-shaped changing trend in trainees’ cognitive control, which differentiates from the subjective evaluations on trainees’ control abilities as shown in Figure 3a-3e. The discrepancy mainly lies in the decrease of cognitive control observed from the Training stage to the PracticeA stage, which may be interpreted by the negative correlation between uncertainty and cognitive control. The PracticeA stage could be associated with increased uncertainty due to the absence of an instructor’s help when trainees moved from the Training stage to the PracticeA stage. From this standpoint, the PracticeA stage corresponds to a situation of low cognitive control, where trainees do not know when or where to control or intervene to achieve good performance, as discussed in the literature^71,72^. This is similar to the findings from Botvinick et al.’s work^71^, where the role of conflict monitoring was emphasized as a key component in cognitive control, particularly in situations where there is ambiguity or uncertainty in decision-making processes. The anterior cingulate cortex (ACC) is central to detecting conflict in information processing, which then triggers adjustments in cognitive control to optimize performance. The drop in cognitive control during the PracticeA stage observed in our analysis results could be

Overall, our STAGE comparisons under Baseline tasks indicated that trainees’ cognitive control may have slightly changed under varied stages within a pilot training process considering the lack of significant changes across the tested EEG features. However, the subjective assessments demonstrated enhanced control performance along the tested pilot training process, including significant cognitive improvements in heading, altitude, and rate of climb/descent (Figure 3a-3c), as well as control management in roll and pitch (Figure 3d and 3e) from Training to PracticeA and from Training to PracticeB while conducting the same Baseline tasks. Such improvements in trainees’ cognitive control were also supported by the significant increases in theta band power from the Training stage to PracticeA stage (over central, temporal, parietal, and occipital sites ) and the significant theta increases from the Training stage to PracticeB stage (over frontal, central, and temporal sites), among the limited significant changes observed in STAGE comparisons. As indicated by the entropy rate, the improvements in cognitive control from PracticeA stage to PracticeB stage could be explained from the skill acquisition point of view, which may contribute to a broad set of cognitive functions that were improved after repeated practice^73,74^. This also aligns with the insight from the study^71^, as cognitive control decreases during uncertain conditions (Training stage), then recovers as trainees become more familiar with the tasks and their requirements from PracticeA to PracticeB. The idea of conflict monitoring could further elaborate on the process by which cognitive control is disrupted by uncertainty and then re-established as trainees adapt. From a different point of view, the inconsistency in the results of microstate features with subjective evaluations can be seen as an opportunity rather than a limitation, which may arise from the complex and multifaceted nature of microstate analysis. In particular, EEG microstate analysis may have the potential to complement subjective evaluations by uncovering hidden patterns or subtle changes in cognitive control that may not be readily apparent to human evaluators. Microstate analysis offers a unique window into the dynamic organization of neural activity, revealing transient brain states that could be crucial for understanding the cognitive processes involved during a pilot training process.

### Trainees’ cognitive control under different stages and varied task demands

Apart from the aforementioned analysis on Baseline tasks (unvaried task demands), the present research also investigated the changes in trainees’ cognitive control under varied task demands. The research question was whether the same changes observed in the analysis of Baseline tasks could be observed if the same analysis was applied to Trial tasks with varied task demands. However, no significant changes were captured by any of the tested features in the STAGE comparisons on Trial tasks.

What can be learned from the similarity and discrepancy between the results obtained on Trial tasks and the aforementioned results obtained on Baseline tasks? The EEG microstate features seemed to be more robust than spectral features in reflecting trainees’ cognitive changes across varied tasks, while the spectral features seemed to be more sensitive to changes in task demands and difficulty levels. During the pilot training process, trainees were instructed to conduct the same maneuvers across the twenty-two Baseline tasks, whereas the maneuvers in the twenty-two Trial tasks varied from one to another with varied difficulty levels and were presented in a pseudo-randomized order. As a result, the cognitive changes captured by certain features under Trial tasks could be triggered by the variations in task demands or difficulty levels, whereas under Baseline tasks the effect of task variations could be avoided. The observed changes under Baseline tasks may be a better reflection of the actual changes in trainees’ cognitive control when the task demands remain stable throughout the pilot training process.

Furthermore, the quantitative assessments, which have been widely applied in current pilot training programs, could serve as the validation of the EEG features in indicating variations in trainees’ cognitive control during the pilot training process even under varied task demands. Among the five evaluated assessment dimensions, skilled performance (altitude) was the only dimension that showed significant increases from Training to PracticeA and also from Training to PracticeB under Trial tasks (Figure 3b). Taking the non-significant changes into consideration, the three skilled performance dimensions (heading, altitude, and rate climb/descent) showed an increasing trend from the Training stage to PracticeA stage and then to PracticeB stage (Figure 3a-3c). Taken together, the subjective evaluations indicated that trainees’ control skills improved and performed better at the confronted flight tasks as the training process continued, which may or may not be reflected in the EEG indices applied in this research. Such improvements in trainees’ cognitive control abilities could not only be captured under unvaried task demands but could also be reflected in tasks with varied demands.

## Limitations and future work

The present research has some limitations that will be addressed in our future work. One limitation lies in the lack of behavioral analysis of trainees’ learning behaviors recorded by cameras. This limitation arises mainly due to the subjectivity involved in behavior analysis. However, we recognize the importance of integrating such behavioral analysis into our research. One future direction is to conduct a reliable trainees’ learning behavior analysis and relate them to the results obtained from physiological measures and subjective assessments. Another limitation is that the research findings reported in the present research were obtained based mainly on EEG-based analysis while other physiological measures were not covered. In our future work, we will continue to explore less intrusive techniques such as ECG and GSR through wearable devices, particularly in studies involving experienced pilots. These methods will allow for more practical and naturalistic assessments of cognitive control, complementing the advantages of EEG in real-world pilot training scenarios. Moreover, we will continue to refine our EEG pre-processing pipeline for our future research, incorporating a comparison of MARA with other advanced machine learning-based artifact rejection methods across multiple datasets to optimize artifact removal and improve data integrity in studies involving complex cognitive tasks. Lastly, but importantly, we plan to expand the dataset by including larger datasets and more diverse participant groups, including experienced pilots, to enhance the robustness and generalizability of the findings.

## Conclusions

The present study aimed to monitor variations in trainees’ cognitive control during a pilot training process using EEG microstate analysis. We conducted a series of comparisons between different flight task types and among different training stages to investigate whether and how pilot trainees’ cognitive control responded to task variations and fluctuated along the pilot training process. By comparing the two types of tasks, our results showed that Trial tasks (differentiated tasks) were associated with reduced cognitive control compared to Baseline tasks (repetitive tasks), which was consistent with our hypothesis about the relationship between cognitive control and two different task types. When confronted with novel and unfamiliar task assignments, trainees may perceive higher uncertainty and stress than when dealing with familiar tasks. Besides, the applied Trial tasks were designed with larger task demands and higher task difficulty in our pilot training experiment. These results also align with the research findings on decreased cognitive control triggered by the presence of novel material in the literature. Moreover, the same EEG quantities were also applied in comparisons among three different stages to monitor how trainees’ cognitive control would change along the pilot training process. Our comparison results on Baseline tasks indicated that trainees’ cognitive control varied across different training stages within the tested simulator-based pilot training process. When compared to the subjective expert assessments, the reported consistency and inconsistency between certain EEG features with the subject evaluations suggest both the applicability of integrating EEG quantities into the current evaluation system and the potential of using EEG microstate analysis to reveal the hidden cognitive patterns involved during a pilot training process. Additionally, cognitive control variations during the pilot training process could be captured by EEG microstate features, even under varied task demands (Trial condition). The potential contributions of the research include improving the effectiveness of pilot training processes with neurophysiological feedback and developing reliable objective assessments for skilled performance.

## Supplementary Information

Supplementary Information.

## Data Availability

The datasets analyzed during the current study are available from the corresponding author upon reasonable request.

## Code availability

The codes used for EEG microstate analysis are made publicly available in the G-Node repository as follows: https://doi.gin.g-node.org/10.12751/g-node.dh7zhl/.
