# Supplementary material for: Monitoring pilot trainees’ cognitive control under a simulator-based training process with EEG microstate analysis
Source: Sci Rep. 2024 Oct 20;14:24632. doi: 10.1038/s41598-024-76046-0 (PMC11491450; doi:10.1038/s41598-024-76046-0)
Supplement: Supplementary file 1 — Supplementary Information. [file 41598_2024_76046_MOESM1_ESM.pdf]

I. Demographic information details

Table S1 Demographic characteristics of he study participants (N=24)

Characteristic	Category	Number of Participants (n)	Percentage
**Age Group**	21-30 years	16	66.7%
	31-40 years	7	29.2%
	41-50 years	1	4.2%
**Gender**	Female	13	54.2%
	Male	11	45.8%
**Education Level**	High school diploma	3	12.5%
	Bachelor's degree	9	37.5%
	Master's degree	12	50.0%
**Handedness**	Right-handed	22	91.7%
	Left-handed	2	8.3%
**Previous experience**	Experience with any type of flight simulator	7	29.2%
	No Prior Experience	17	70.8%

Data Collection Procedure:

- **Demographic Questionnaire:** Before commencing the experimental tasks, participants completed a questionnaire capturing demographic information, including age, gender, education level, handedness, and prior flight experience.
- **Confidentiality Assurance:** Participants were assured that all personal data would be kept confidential and used solely for research purposes.

Ethical Considerations:

- The experimental protocol was approved by both the Concordia Human Research Ethics Committee and the research ethics board of the National Research Council Canada.
- Participants were informed of their right to withdraw from the study at any time without penalty.

This section provides detailed demographic information about the participants involved in the study, ensuring transparency and enabling readers to understand the composition of the sample. It adheres to ethical standards by maintaining participant confidentiality and providing all necessary information for reproducibility and further analysis.

II. Pre-processing details on bad channels and components

Table S2 Total number of bad channels identified and removed based on the applied criteria

Session	1	2	3	4	5	6	7	8	9	10	11	12	13	14	15	16	17	18	19	20	21	22
P01	12	7	13	10	11	13	9	1	1	1	3	0	0	1	1	0	1	1	1	1	2	2
P02	3	3	2	2	2	2	3	1	5	4	2	2	2	2	3	1	1	2	0	3	1	1
P03	5	5	4	4	3	4	4	6	5	4	4	4	3	4	6	4	6	5	4	6	4	3
P04	3	5	6	4	3	7	7	3	6	3	3	3	1	11	3	3	4	2	6	5	4	2
P05	7	7	10	6	4	5	2	3	4	2	10	7	1	7	3	6	3	7	5	4	3	6
P06	0	7	1	3	6	0	1	0	0	2	0	0	0	1	0	8	4	9	6	5	1	5
P07	1	2	2	4	3	1	4	3	2	7	2	1	2	2	4	5	0	5	6	0	5	4
P08	7	5	3	7	4	8	6	8	6	4	8	4	2	6	8	6	6	6	6	4	7	3
P09	2	1	5	4	0	3	0	1	1	8	1	1	3	0	1	4	5	2	2	1	2	3
P10	7	4	7	6	7	5	6	8	3	6	4	2	8	7	4	9	2	6	0	1	1	3
P11	3	4	7	6	6	5	5	5	1	3	4	6	3	5	5	2	5	3	7	7	3	1
P12	5	3	5	4	5	4	5	7	4	6	5	2	4	4	5	5	4	2	4	3	5	4
P13	1	1	1	2	1	2	1	0	0	0	1	0	0	0	0	3	3	3	3	3	5	6
P14	6	5	3	4	2	6	2	3	5	8	5	2	8	2	5	2	5	2	5	4	2	2
P15	4	1	5	5	1	2	3	4	2	6	5	5	8	8	6	5	12	10	1	8	4	3
P16	2	1	3	3	1	4	1	3	1	1	1	1	2	4	1	2	1	1	5	3	5	2
P17	7	7	2	3	4	4	3	6	4	3	3	3	6	5	5	8	5	4	2	6	6	2
P18	5	1	1	1	1	1	1	0	0	7	0	2	1	2	8	5	8	1	1	1	1	1
P19	0	4	2	2	1	4	3	5	8	2	8	2	3	1	5	2	5	6	4	2	6	2
P20	8	6	11	9	7	10	6	4	3	6	3	4	5	3	1	4	5	0	2	3	2	6
P21	6	9	10	8	4	7	11	6	4	7	8	6	5	7	6	4	4	9	2	7	5	7
P22	2	4	1	5	0	1	2	3	0	3	2	7	3	1	3	7	0	5	6	7	2	7
P23	4	1	5	4	2	2	3	3	4	5	5	3	3	4	2	4	2	3	6	1	7	1
P24	3	2	2	3	2	7	1	5	3	2	2	1	2	1	4	4	2	1	5	4	8	1

Table S3 Number of remaining components after artifact removal using MARA

Session	1	2	3	4	5	6	7	8	9	10	11	12	13	14	15	16	17	18	19	20	21	22
P01	32	35	34	35	32	29	33	32	33	36	29	35	27	30	30	35	37	36	35	35	33	25
P02	35	34	42	36	41	36	42	35	39	38	37	41	37	38	36	40	37	30	38	39	32	43
P03	47	48	37	43	44	40	45	46	47	45	43	43	40	41	44	46	45	39	41	40	41	39
P04	37	36	31	36	34	37	32	30	37	39	40	36	44	33	39	36	34	41	39	40	39	40
P05	21	14	16	15	20	20	14	10	21	17	29	28	15	25	14	28	11	15	21	21	11	32
P06	41	38	47	43	43	42	35	45	41	39	37	38	37	34	39	45	44	39	41	41	37	38
P07	16	14	19	18	20	24	22	25	32	40	26	21	29	20	36	32	27	33	35	25	27	25
P08	47	48	50	43	51	49	40	49	50	54	49	54	46	51	49	53	50	48	56	51	51	41
P09	40	38	30	46	33	37	37	36	30	42	37	26	38	40	43	43	39	40	34	34	42	36
P10	36	27	41	37	43	32	40	42	27	35	28	23	40	35	41	46	41	40	51	46	39	41
P11	16	43	38	46	34	35	25	40	33	22	38	37	45	48	45	37	40	44	41	41	38	42
P12	31	36	31	35	39	36	44	38	38	40	36	34	35	29	44	43	39	26	46	43	39	34
P13	41	47	44	41	46	42	44	48	50	52	50	50	55	55	50	54	48	48	47	51	47	54
P14	23	30	32	32	38	29	34	38	35	37	31	35	35	34	40	36	42	31	36	38	41	24
P15	48	55	46	42	51	51	47	47	49	47	43	49	42	47	49	50	45	44	48	43	52	47
P16	31	24	31	28	26	29	27	30	33	29	31	26	27	33	37	38	27	25	33	27	36	34
P17	36	33	39	36	34	36	37	31	31	23	29	31	38	32	31	37	37	33	30	32	36	43
P18	32	32	36	37	37	36	29	47	42	39	41	35	34	31	37	26	31	34	38	31	40	37
P19	36	33	35	27	24	39	41	41	40	25	37	30	31	35	34	26	40	42	36	21	36	29
P20	39	27	32	32	32	32	30	45	43	33	33	32	33	39	37	35	39	42	39	37	33	35

P21	35	29	39	32	38	44	31	43	37	35	41	46	41	41	40	40	27	42	50	46	50	40
P22	39	38	24	42	27	35	37	39	31	34	30	28	30	26	24	31	38	32	31	32	31	34
P23	42	41	40	40	41	38	40	48	36	38	40	41	44	36	40	40	39	36	38	34	35	34
P24	19	23	16	17	20	22	29	23	23	17	20	21	22	16	18	26	21	17	23	28	26	22

III. EEG microstates under different conditions

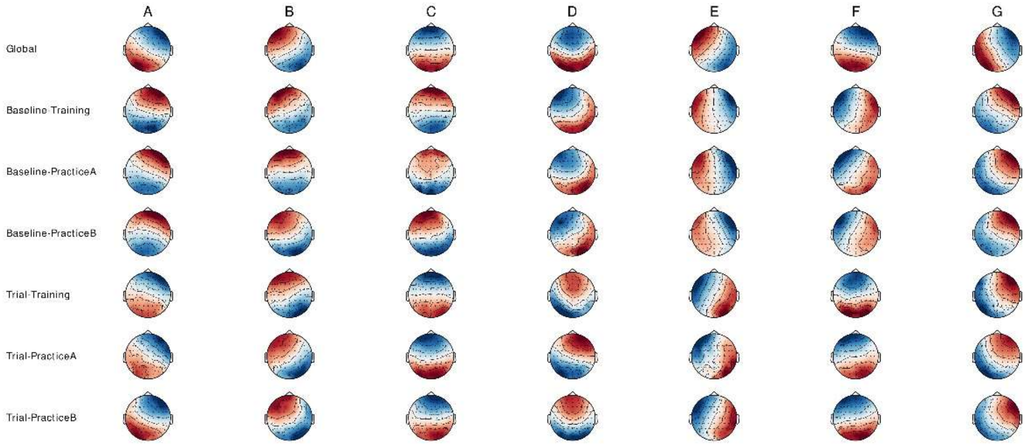

Figure S1 The spatial configuration of the seven microstate classes (A, B, C, D, E, F, and G) across TASK and STAGE (global) and for each task type (Baseline and Trial) during different stages (Training, PracticeA, and PracticeB)

IV. Detailed information of statistical analysis

Table S4 P-values for paired TASK and STAGE comparisons on each quantitative evaluation dimension

Comparison	Dimension				
	P-H	P-A	P-R	C-R	C-P
B-S1 Vs. T-S1	0.021* ↘	0.000*** ↘	0.000*** ↘	0.000*** ↘	0.000*** ↘
B-S2 Vs. T-S2	0.001*** ↘	0.000*** ↘	0.000*** ↘	0.000*** ↘	0.000*** ↘
B-S3 Vs. T-S3	0.002*** ↘	0.000*** ↘	0.000*** ↘	0.000*** ↘	0.000*** ↘
B-S1 Vs. B-S2	0.072	0.017* ↗	0.039* ↗	0.148	0.016* ↗
B-S1 Vs. B-S3	0.022* ↗	0.021* ↗	0.006 ** ↗	0.009** ↗	0.002*** ↗
B-S2 Vs. B-S3	0.311	1.000	1.000	0.230	1.000
T-S1 Vs. T-S2	1.000	0.046* ↗	1.000	0.463	1.000
T-S1 Vs. T-S3	0.567	0.001*** ↗	0.942	1.000	1.000
T-S2 Vs. T-S3	1.000	0.066	0.949	0.130	1.000

* $\rho \leq 0.050$, ** $\rho \leq 0.010$, *** $\rho \leq 0.005$
↗ Quantitative assessment increases
↘ Quantitative assessment decreases

Table S5 P-values for paired TASK and STAGE comparisons in theta band power

Comparison	Area				
	Frontal	Central	Temporal	Parietal	Occipital
B-S1 Vs. T-S1	0.094	0.007** ↘	0.086	0.000*** ↗	0.000*** ↗
B-S2 Vs. T-S2	0.000*** ↘	0.000*** ↘	0.000*** ↘	0.000*** ↗	0.000*** ↗
B-S3 Vs. T-S3	0.000*** ↘	0.000*** ↘	0.003*** ↘	0.001*** ↗	0.000*** ↗
B-S1 Vs. B-S2	0.314	0.019 * ↗	0.008 ** ↗	0.034 * ↗	0.020 * ↗
B-S1 Vs. B-S3	0.049 * ↗	0.048 * ↗	0.046 * ↗	0.173	1.000
B-S2 Vs. B-S3	0.317	1.000	1.000	1.000	0.097
T-S1 Vs. T-S2	0.124	0.300	1.000	1.000	1.000
T-S1 Vs. T-S3	0.906	1.000	1.000	0.094	1.000
T-S2 Vs. T-S3	0.828	0.848	1.000	0.174	1.000

* $\rho \leq 0.050$, ** $\rho \leq 0.010$, *** $\rho \leq 0.005$
↗ Theta band power increases
↘ Theta band power decreases

Table S6 P-values of paired TASK and STAGE comparisons on EEG microstate coverage

Comparison	Microstate classes						
	Class A	Class B	Class C	Class D	Class E	Class F	Class G
B-S1 Vs. T-S1	0.606	0.018* ↗	0.269	0.000*** ↗	0.000*** ↘	0.084	0.000*** ↘
B-S2 Vs. T-S2	0.218	0.030* ↗	0.014* ↗	0.000*** ↗	0.000*** ↘	0.007** ↗	0.000*** ↘
B-S3 Vs. T-S3	0.924	0.091	0.100	0.000*** ↗	0.000*** ↘	0.002*** ↗	0.000*** ↘
B-S1 Vs. B-S2	0.920	0.987	0.377	0.150	1.000	0.337	1.000
B-S1 Vs. B-S3	1.000	0.806	0.721	1.000	1.000	0.228	0.451
B-S2 Vs. B-S3	1.000	1.000	1.000	0.097	0.475	1.000	0.521
T-S1 Vs. T-S2	1.000	0.944	1.000	1.000	1.000	0.165	0.672
T-S1 Vs. T-S3	0.585	0.723	0.710	1.000	0.606	0.315	0.086
T-S2 Vs. T-S3	1.000	1.000	1.000	0.634	0.507	1.000	1.000

* $\rho \leq 0.050$, ** $\rho \leq 0.010$, *** $\rho \leq 0.005$
↗ Microstate coverage increases
↘ Microstate coverage decreases

Table S7 P-values of paired TASK and STAGE comparisons on EEG microstate occurrence

Comparison	Microstate classes						
	Class A	Class B	Class C	Class D	Class E	Class F	Class G
B-S1 Vs. T-S1	0.435	0.011* ↗	0.012* ↗	0.000*** ↗	0.024* ↘	0.011* ↗	0.004*** ↘
B-S2 Vs. T-S2	0.726	0.006** ↗	0.005*** ↗	0.000*** ↗	0.002*** ↘	0.002*** ↗	0.000*** ↘
B-S3 Vs. T-S3	0.085	0.013* ↗	0.018* ↗	0.000*** ↗	0.005*** ↘	0.000*** ↗	0.003*** ↘
B-S1 Vs. B-S2	1.000	1.000	1.000	0.157	1.000	0.690	0.377
B-S1 Vs. B-S3	1.000	0.627	0.724	1.000	1.000	0.225	0.748
B-S2 Vs. B-S3	0.377	0.530	1.000	0.023* ↘	1.000	0.790	1.000
T-S1 Vs. T-S2	1.000	1.000	1.000	1.000	1.000	0.298	0.521
T-S1 Vs. T-S3	1.000	0.806	1.000	1.000	1.000	0.551	0.400
T-S2 Vs. T-S3	1.000	0.636	1.000	0.287	1.000	1.000	1.000

* $\rho \leq 0.050$, ** $\rho \leq 0.010$, *** $\rho \leq 0.005$
↗ Microstate occurrence increases
↘ Microstate occurrence decreases

Table S8 P-values of paired TASK and STAGE comparisons on EEG microstate duration

Comparison	Microstate classes						
	Class A	Class B	Class C	Class D	Class E	Class F	Class G
B-S1 Vs. T-S1	0.041* ↘	0.693	0.904	0.000*** ↗	0.000*** ↘	0.583	0.000*** ↘
B-S2 Vs. T-S2	0.030* ↘	0.380	0.263	0.000*** ↗	0.000*** ↘	0.611	0.000*** ↘
B-S3 Vs. T-S3	0.041* ↘	0.532	0.913	0.000*** ↗	0.000*** ↘	0.516	0.000*** ↘
B-S1 Vs. B-S2	1.000	1.000	0.728	0.222	1.000	0.556	1.000
B-S1 Vs. B-S3	0.717	1.000	1.000	0.563	1.000	0.830	0.628
B-S2 Vs. B-S3	1.000	1.000	1.000	1.000	0.598	1.000	0.292
T-S1 Vs. T-S2	0.960	0.393	1.000	1.000	1.000	0.415	0.911
T-S1 Vs. T-S3	0.339	0.984	0.861	1.000	0.485	0.898	0.221
T-S2 Vs. T-S3	0.543	1.000	1.000	1.000	0.213	1.000	0.962

* $\rho \leq 0.050$, ** $\rho \leq 0.010$, *** $\rho \leq 0.005$
↗ Microstate duration increases
↘ Microstate duration decreases

Table S9 P-values for paired TASK and STAGE comparisons in entropy rate.

Comparison	Entropy rate
B-S1 Vs. T-S1	0.000*** ↘
B-S2 Vs. T-S2	0.000*** ↘
B-S3 Vs. T-S3	0.000*** ↘
B-S1 Vs. B-S2	0.426
B-S1 Vs. B-S3	1.000
B-S2 Vs. B-S3	0.039* ↘
T-S1 Vs. T-S2	1.000
T-S1 Vs. T-S3	1.000
T-S2 Vs. T-S3	0.492

* $\rho \leq 0.050$, ** $\rho \leq 0.010$, *** $\rho \leq 0.005$
↗ Entropy rate increases
↘ Entropy rate decreases

Table S10 P-values for paired TASK and STAGE comparisons in Hurst exponent

Comparison	Hurst exponent
B-S1 Vs. T-S1	0.000*** ↘
B-S2 Vs. T-S2	0.000*** ↘
B-S3 Vs. T-S3	0.000*** ↘
B-S1 Vs. B-S2	0.192
B-S1 Vs. B-S3	1.000
B-S2 Vs. B-S3	0.270
T-S1 Vs. T-S2	1.000
T-S1 Vs. T-S3	1.000
T-S2 Vs. T-S3	0.346

* $\rho \leq 0.050$, ** $\rho \leq 0.010$, *** $\rho \leq 0.005$
↗ Hurst exponent increases
↘ Hurst exponent decreases

V. Performance comparison between two groups

Table S11 Permutation test on experience effect

Dimension		D1	D2	D3	D4	D5
Permutation test	Observed difference	-0.16616	-0.33117	-0.07983	-0.23568	0.07410
	p-value	0.2092	0.12890	0.73970	0.10660	0.71210
